# Effect of osteosarcopenia on feeding status in hospitalized patients with suspected dysphagia

**DOI:** 10.1371/journal.pone.0315091

**Published:** 2024-12-19

**Authors:** Midori Miyagi, Hideki Sekiya, Satoru Ebihara

**Affiliations:** 1 Department of Rehabilitation Medicine, Tohoku University Graduate School of Medicine, Sendai, Japan; 2 Department of Oral Surgery, School of Medicine, Toho University, Tokyo, Japan; Gyeongsang National University, REPUBLIC OF KOREA

## Abstract

**Objectives:**

Osteosarcopenia is a combination of sarcopenia and osteoporosis that increases mortality rates among older people compared with either alone. This study aimed to identify the contribution of osteosarcopenia to the development and severity of dysphagia.

**Methods:**

We retrospectively reviewed the medical charts of 211 patients aged ≥ 65 years who were referred to the dysphagia rehabilitation team. Based on Functional Oral Intake Scale (FOIS) scores, we classified the patients with (FOIS scores 1–5) and without (FOIS scores 6, 7) dysphagia as Type A and those with (FOIS scores 1, 2) and without (FOIS score 3–7) enteral feeding as Type B. Based on chest computed tomography (CT) findings we then defined patients with T4 (MI) and pectoralis (PMI) muscle indexes, L1 attenuation, and T4MI, PMI, and L1 attenuation below the cutoff values as having sarcopenia, osteoporosis, and osteosarcopenia, respectively.

**Results:**

The FOIS scores were significantly lower among patients with osteosarcopenia than among those without sarcopenia or osteoporosis. Moreover, PMI and FOIS scores significantly and positively correlated, and PMI was significantly lower in the group with, than without, enteral feeding. Osteoporosis and osteosarcopenia were significant in the patients who were fed enterally (p = 0.032 and 0.047, respectively).

**Conclusions:**

Patients with sarcopenia and osteoporosis undergoing swallowing rehabilitation tended to have severe dysphagia that required much medical attention.

## Introduction

Various factors in older adults can cause dysphagia that leads to malnutrition and aspiration pneumonia. Sarcopenia is the age-related loss of skeletal muscle mass and strength that includes muscles associated with swallowing. Sarcopenia has become a significant concern among older adults because it reduces not only the ability to participate in activities of daily living (ADLs) but also life expectancy [1]. Sarcopenic dysphagia has a worse recovery rate than dysphagia resulting from other pathological states [2].

Osteoporosis in ~100 million adults worldwide increases the risk of fractures and mortality, and diminishes the ability to manage ADLs [3, 4]. Furthermore, older adults with osteoporosis-associated femoral and vertebral fractures who develop dysphagia have significantly lower skeletal muscle indexes (SMI) and bone mineral density (BMD) than those who do not [5]. These findings suggested that both sarcopenia and bone quality are associated with dysphagia in older adults.

The recently proposed term, osteosarcopenia, contributes to a reduced ability to manage ADLs and increased mortality rates among older people compared with either sarcopenia or osteoporosis alone [6]. Although this suggests that osteosarcopenia profoundly affects dysphagia in older adults, it has not been confirmed.

Sarcopenia is diagnosed according to the European Working Group on Sarcopenia in Older People [7] and the Asian Working Group for Sarcopenia 2019 criteria [8] that includes assessments of the quality, output, and quantity of skeletal muscle. Dual-energy X-ray absorptiometry (DXA) is the gold standard for assessing skeletal muscle mass to diagnose or rule out sarcopenia. However, bioelectrical impedance analysis (BIA) has also recently become widespread in clinical practice.

Sarcopenia can be indirectly determined on chest computed tomography (CT) images by quantifying the skeletal muscle cross-sectional area (CSA) on a single slice at the level of the third lumbar vertebra (L3). Lean areas in erector spinae muscles are measured in Hounsfield units (HU). Skeletal muscle mass at the T4 level has also been analyzed using chest CT. Quantifying the skeletal muscle mass of the thorax and in various diseases is important [9–12].

Cutoff values for thoracic skeletal muscles in men and women have been established to diagnose sarcopenia [13]. Furthermore, the quality of L1 trabecular bone assessed by CT in patients with breast cancer identified < 90 HU as an independent factor for the development of fractures closely correlated with T-scores below -2.5 on DXA, which is the World Health Organization reference for osteoporotic disease [14].

We quantified the pectoralis major muscle, T4 trans-skeletal muscle area, and L1 vertebral trabecular bone attenuation based on chest CT imaging data to identify sarcopenia, osteoporosis, and osteosarcopenia and their contribution to the development and severity of dysphagia in older adults.

## Material and methods

### Patients

We retrospectively reviewed the medical charts of 211 patients aged ≥ 65 years who were referred to the team for dysphagia rehabilitation at Toho University Omori Medical Center between April 2019 and March 2022. We collected information about demographic information, etiology of admission, and comorbidities of patients who were hospitalized at the center and screened for dysphagia. Those who tested positive underwent a further a two-step screen. Oral function was determined, and repetitive saliva swallowing was tested in the first step. The ability of patients who tested negative in the first step to swallow jelly and thickened liquids was screened in the second step. Patients who tested positive at each step were referred to the dysphagia rehabilitation team. Accredited and experienced nurses in charge of dysphagia in each ward conducted the two-step screening. These nurses also work with those in the dysphagia rehabilitation team who are accredited by the Japanese Society of Dysphagia Rehabilitation. Patients were directly referred to the dysphagia rehabilitation team if an attending physician suspected dysphagia. This team was established in 2005 to prevent aspiration, dysphagia, and choking among hospitalized patients. The team comprises dentists, otolaryngologists, rehabilitation physicians, nurses who specialize in dysphagia, speech therapists, registered dietitian nutritionists, dental hygienists and pharmacists.

**Fig 1:**
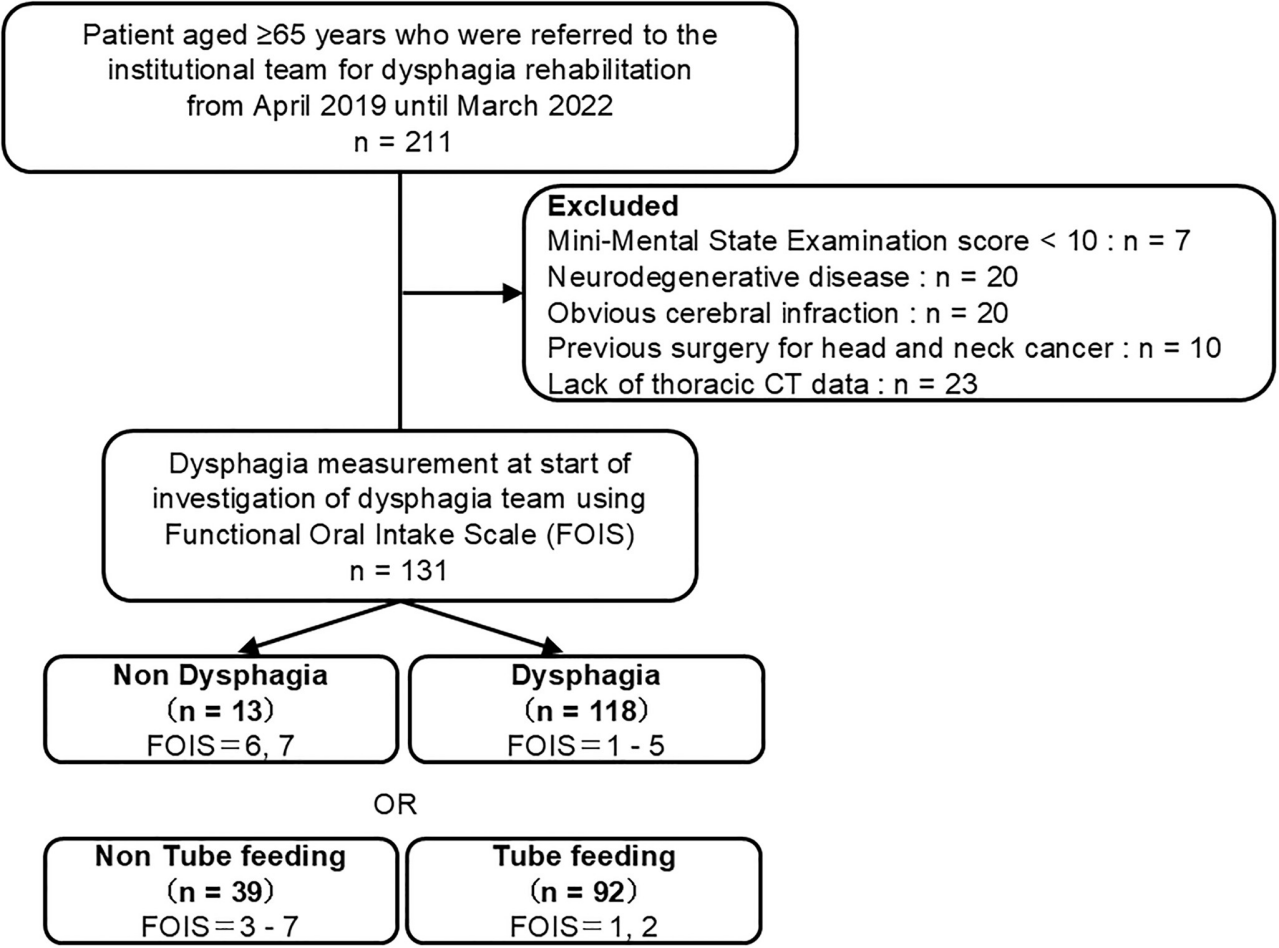
Flow of patients through the study.

Patients with severe dementia (Mini-Mental State Examination score < 10) (n = 7), neurodegenerative disease (n = 20), obvious cerebral infarction (n = 20), previous surgery for head and neck cancer (n = 10) as previous history, or without thoracic CT data (n = 23) were excluded from this study. We finally analyzed data from 131 patients (Fig 1). Based on our calculations, a minimal sample size of 128 participants was required to achieve a significance level of 5%, power of 80%, and effect size of 0.5.

The Ethics Committee at Toho University Omori Medical Center approved the study protocol (No. M22112) and informed consent was obtained from all participants in the study using an opt-out process. All data of patients were accessed for research purpose on 3^rd^ August 2022. And all data were anonymized before being accessed by researchers.

### Dysphagia evaluation

The dysphagia rehabilitation team conducted meal rounds, and the rehabilitation physician prescribed training with a speech therapist. The severity of dysphagia was assessed using fiberoptic endoscopic and/or videofluoroscopic swallowing studies and the Functional Oral Intake Scale (FOIS) [15]. At the Toho University Omori Medical Center, the type of meal given to patients with dysphagia was determined by the consensus of a dentist, speech therapist, nurse specializing in dysphagia, rehabilitation physician, registered dietitian nutritionists and an otolaryngologist. The reference for determining the type of meal was based on the best ability of patients with dysphagia to swallow. Patients with FOIS scores from 1 to 7 were respectively defined as [15] no intake of food or liquids by mouth, tube-dependent with minimal attempts to swallow food or liquid, tube-dependent with consistent oral food or liquid intake, a consistent total oral diet, total oral diet with multiple consistencies but requiring special preparation or compensations; total oral diet with multiple consistencies without special preparation but with specific food limitations, and total oral diet with no restrictions. We defined FOIS scores of 1–5 and 6 and 7 as with and without dysphagia (Type A), respectively, and FOIS scores of 1 and 2, and 3–7 as respectively requiring enteral feeding or not (Type B).

#### Determination of sarcopenia, osteoporosis, and osteosarcopenia using CT

All patients were assessed using a SOMATOM CT scanner (Siemens Healthineers, Erlangen, Germany). Images were acquired at slice intervals of 1–5 mm using SOMATOM Definition Flash, SOMATOM Definition AS+, or SOMATOM Definition Edge according to Digital Imaging and Communications in Medicine (DICOM) specifications.

Imaging data were analyzed using SYNAPSE VINCENT (Fujifilm Medical Systems, Tokyo, Japan) CT software.

We obtained CSAs of the pectoralis, intercostalis, paraspinal, serratus, and latissimus muscles at T4 (T4_CSA_) and that of the pectoralis muscle area (PM_CSA_) at the fourth vertebral region.

We defined the middle of the fourth thoracic vertebra as the T4 slice. Reviewers identified single cross-sectional images. The CSA in slices was applied with a pixel attenuation of -30 to +150 HU of skeletal muscle, and the boundaries between different tissues were corrected using SYNAPSE. We initially quantified T4_CSA_ and PM_CSA_ using SYNAPSE (S1A and S1B Fig). The T4 muscle index (T4MI) was then calculated as T4_CSA_ divided by height squared, and the pectoralis muscle index (PMI) was calculated as the PM_CSA_ divided by height squared. The cutoff values for PMI and T4MI were 10.17 and 33.69 cm^2^/m^2^ in men, and 7.31 and 26.01 cm^2^/m^2^ in women, respectively. S1 Table shows the cutoff values [13, 14].

We measured the mean trabecular attenuation of the appropriate L1 slice by placing an ovoid region of interest (ROI) within the anterior–superior portion of the trabecular space avoiding the cortical bone and focal sclerotic or lytic lesions using SYNAPSE (S1C Fig). Patients with L1 attenuation ≤ 90 HU were defined as having osteoporosis [14, 16].

Based on these measurements and indices, we determined that patients with T4MI and PMI, L1 attenuation, and T4MI, PMI, and L1 attenuation values below the relevant cutoff values had sarcopenia, osteoporosis, and osteosarcopenia, respectively.

All CT images were independently acquired by MM and SE who were blinded to the clinical information of the patients. We used averaged PMI, T4MI, and L1 attenuation values. Moreover, we analyzed inter-rater-reliability.

### Statistical analysis

Values are expressed as means ± standard deviation (SD) and range. Differences in baseline characteristics were analyzed using two-tailed t-tests for continuous variables, Mann–Whitney *U* tests for non-normally distributed variables, and χ^2^ tests for categorical variables.

Whether FOIS scores correlated with osteosarcopenia factors was evaluated using Spearman correlation coefficients. Patients with sarcopenia, osteoporosis, and osteosarcopenia were compared using one-way analysis of variance (ANOVA) and Games–Howel tests. Ordinal logistic regression analyses with forward selection were used to evaluate the association between FOIS and age, PMI, presence or absence of lung cancer, presence or absence of pulmonary disease, and presence or absence of osteoporosis. Interclass correlation coeffcients {ICC [2,1]} for intra-observer and inter-observer errors were analyzed. Values with p < 0.05 were considered statistically significant. All data were analyzed using SPSS 17 (SPSS Inc., Chicago, IL, USA).

## Result

### Characteristics of patients

Table 1 shows the clinical and demographic characteristics of the 131 patients.

**Table 1:** Patient characteristics and comparison of patients with or without dysphagia.

	Total	No dysphagia n = 13	Dysphagia n = 118	p
Age (y)	80.1 ± 7.25	76.1 ± 7.9	80.5 ± 7.1	ns^a^
Female/Male	50 / 81	4 / 9	46 / 72	ns^b^
BMI (kg/m^2^)	1.58 ± 0.09	21.1 ± 4.5	20.6 ± 4.3	ns^a^
Etiology				
Pulmonary disease	40	0	40	0.01
Cardiac disease	30	2	28	ns^b^
Cancer (without lung)	13	2	11	ns^b^
Lung cancer	11	6	5	<0.001
Dermatosis	6	2	4	ns^b^
Digestive system disease	6	0	6	ns^b^
Renal urological disease	6	0	6	ns^b^
Endocrine disease	5	0	5	ns^b^
Meningitis	2	0	2	ns^b^
Orthopedic disease	2	1	1	ns^b^
Psychiatric disease	2	0	2	ns^b^
Others	8	0	8	ns^b^
Comorbidities (n)				
Hypertension	47	5	42	ns^b^
Diabetes mellitus	36	2	34	ns^b^
Cerebral vascular disease	23	2	21	ns^b^
Chronic kidney disease	14	2	8	ns^b^
Cognitive disorder	11	2	12	ns^b^
Heart failure	10	0	11	ns^b^
Cancer	10	0	10	ns^b^
COPD	9	1	8	ns^b^
Collagen disease	5	1	4	ns^b^
Others	8	1	7	ns^b^
Image measurements				
PM_CSA_ (cm^2^)	24.7 ± 9.3	25.6 ± 9.0	24.6 ± 9.3	ns^a^
T4_CSA_ (cm^2^)	70.9 ± 22.8	67.8 ± 25.4	71.2 ± 22.5	ns^a^
PMI (cm^2^/m^2^)	9.6 ± 3.6	10.3 ± 2.5	9.8 ± 3.6	ns^a^
T4MI (cm^2^/m^2^)	28.3 ± 78.6	27.2 ± 7.6	28.4 ± 8.7	ns^a^
L1 attenuation (HU)	99.8 ± 45.1	115.6 ± 42.7	98.1 ± 45.0	ns^a^

Values are shown as means ± standard deviation or number.

^a^Mann–Whitney U test;

^b^χ^2^ test.

BMI, body mass index; COPD, chronic pulmonary disease; HU, Hounsfield units; PMI, pectoralis muscle index; T4MI, T4 muscle index.

The mean age, height, weight, and body mass index were 80.1 ± 7.25 years, 158.9 ± 8.6 cm, 49.2 ± 9.3 kg, and 20.6 ± 4.3 kg/m^2^, respectively. Etiology of admission was pulmonary disease (n = 40), cardiac disease (n = 30), cancer (without lung) (n = 13), lung cancer (n = 11), dermatosis (n = 6), digestive system disease (n = 6), renal urological disease (n = 6), endocrine disease (n = 5), meningitis (n = 2), orthopedic disease (n = 2), psychiatric disease (n = 2), and others (n = 8). We identified patients with sarcopenia (n = 53), osteoporosis (n = 52), osteosarcopenia (n = 19), and neither sarcopenia nor osteoporosis (n = 20) based on the CT cutoff values.

### Patients with sarcopenia, osteoporosis, osteosarcopenia, and neither sarcopenia nor osteoporosis

We investigated associations between FOIS scores and PMI, T4MI, and L1 attenuation. The ICCs [2, 1] for intra-observer and inter-observer errors 0.97 (0.90, 0.99) and 0.875 (0.60, 0.97) for T4, 0.99 (0.96, 0.10) and 0.92 (0.70, 0.98) for PM, and 0.99 (0.96, 0.10) and 0.95 (0.82, 0.99) for L1, respectively. No significant differences in each parameter were found between MM and SE.

**Fig 2:**
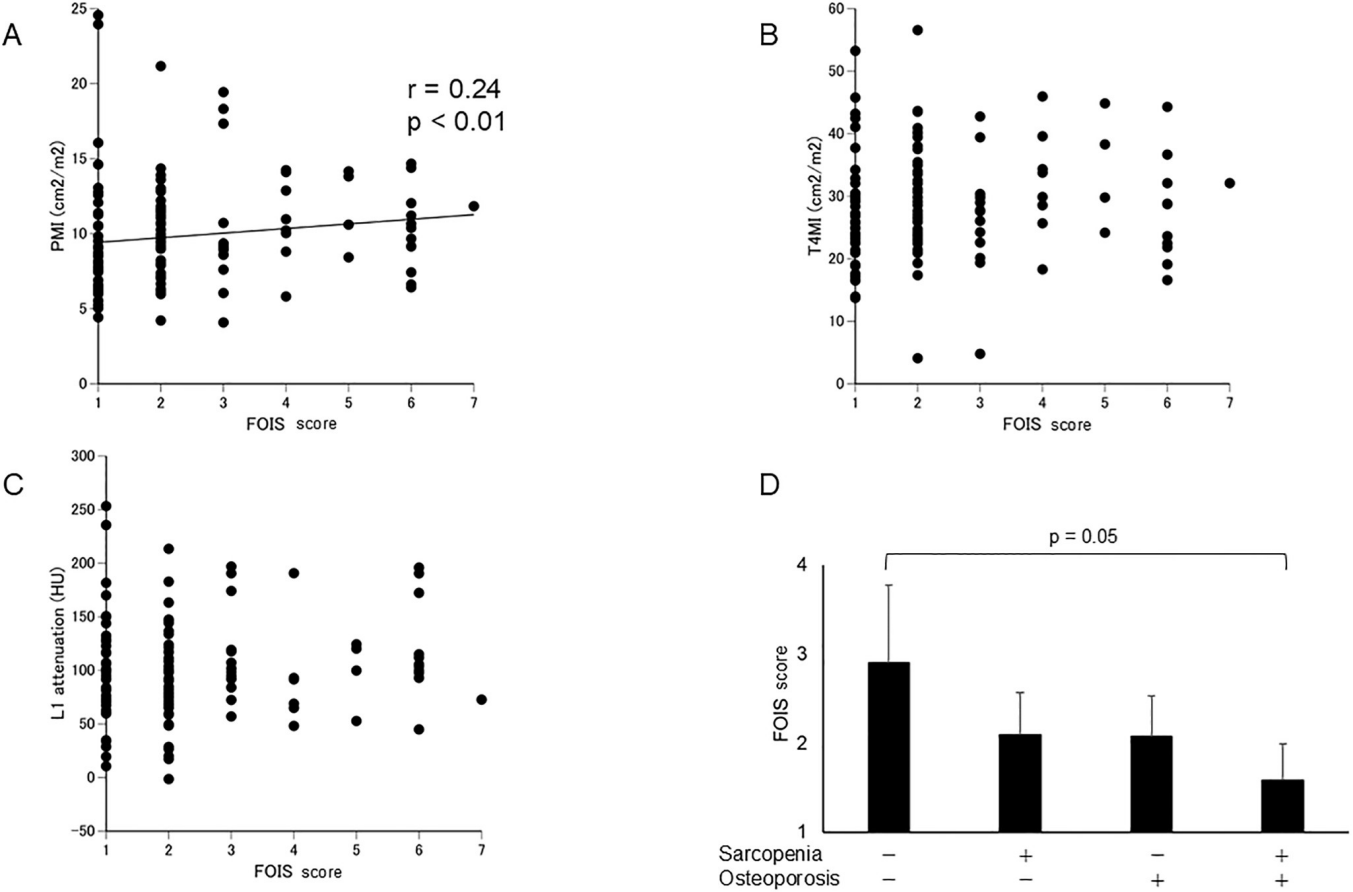
Scatter Plots of Associations and FOIS Scores Scatter diagrams show associations between FOIS scores and (A) PMI, (B) T4MI, and (C) L1 attenuation. (D) FOIS scores among patients with sarcopenia, osteoporosis, and osteosarcopenia. FOIS, Functional Oral Intake Scale; PMI, pectoralis muscle index; T4MI, T4 muscle index.

We found that FOIS scores correlated positively with PMI (p < 0.01, r = 0.24). but not significantly with T4MI and L1 attenuation (Fig 2A–2C). The FOIS score was significantly lower in the group with osteosarcopenia than in those without sarcopenia, or osteoporosis, or osteoporosis alone (Fig 2D, p = 0.05).

### Type A patients with vs. without dysphagia

We found that 118 (90%) Type A patients had dysphagia (Table 1). Regarding etiology, pulmonary disease and lung cancer were associated with the incidence of dysphagia. Age, sex, comorbidities, and imaging findings did not significantly differ between the groups. Sarcopenia, osteoporosis, and osteosarcopenia were not associated with the incidence of dysphagia (Table 2).

**Table 2:** Comparison between patients with (+) and without (-) dysphagia.

		Dysphagia		p^a^
		+ (n = 118)	− (n = 13)	
Sarcopenia (T4MI, PMI)	+	49	4	0.453
	−	69	9	
Osteoporosis	+	50	2	0.059
	−	68	11	
Osteosarcopenia (T4MI PMI L1)	+	19	0	0.117
	−	99	13	

^a^χ^2^ test.

PMI, pectoralis muscle index; T4MI, T4 muscle index.

### Comparison of Type B patients with and without enteral feeding

We found that 92 (70.2%) patients had dysphagia (Table 3). The T4MI, PMI, and L1 attenuation were 28.3 ± 8.6 cm^2^/m^2^, 9.6 ± 3.6 cm^2^/m^2^, and 99.8 ± 45.1 HU, respectively. Table 3 shows a comparison of imaging values between patients with and without enteral feeding. Regarding etiology, lung cancer was associated with the incidence of enteral feeding. The PMI was significantly lower in the group with, than without, enteral feeding (p = 0.04). Osteoporosis and osteosarcopenia were significantly more prevalent in the group with enteral feeding (p = 0.032 and 0.047, respectively; Table 4).

**Table 3:** Comparison between patients with and without enteral feeding.

	Without n = 39	With n = 92	p
Age (y)	79.5 ± 7.9	80.3 ± 6.9	ns^a^
Female/Male	11 / 28	39 / 53	ns
BMI (kg/m^2^)	21.3 ± 3.6	20.4 ± 4.5	ns^a^
Etiology			
Pulmonary disease	10	30	ns^b^
Cardiac disease	8	22	ns^b^
Cancer (without lung)	5	8	ns^b^
Lung cancer	7	4	0.016
Dermatosis	3	3	ns^b^
Digestive system disease	1	5	ns^b^
Renal urological disease	0	6	ns^b^
Endocrine disease	0	5	ns^b^
Meningitis	1	1	ns^b^
Orthopedic disease	1	1	ns^b^
Psychiatric disease	1	1	ns^b^
Others	2	6	ns^b^
Comorbidities (n)			
Hypertension	14	33	ns^b^
Diabetes mellitus	8	28	ns^b^
Cerebral vascular disease	6	17	ns^b^
Chronic kidney disease	3	7	ns^b^
Cognitive disorder	3	11	ns^b^
Heart failure	3	8	ns^b^
Cancer	3	7	ns^b^
COPD	3	6	ns^b^
Collagen disease	2	3	ns^b^
Others	1	7	ns^b^
Image measurements			
PM_CSA_ (cm^2^)	26.6 ± 9.2	23.9 ± 9.2	ns^a^
T4_CSA_ (cm^2^)	72.1 ± 23.9	70.3 ± 22.3	ns^a^
PMI (cm^2^/m^2^)	10.6 ± 3.4	9.5 ± 3.6	0.04^a^
T4MI (cm^2^/m^2^)	28.7 ± 8.5	28.1 ± 8.6	ns^a^
L1 attenuation (HU)	108.5 ± 41.6	96.2 ± 46.0	ns^a^

Values are shown as means ± standard deviation or number.

^a^Mann–Whitney U test;

^b^χ^2^ test.

BMI, body mass index; COPD, chronic pulmonary disease; HU, Hounsfield units; PMI, pectoralis muscle index; T4MI, T4 muscle index.

**Table 4:** Comparison between patients with (+) and without (-) enteral feeding.

		Enteral feeding		p^a^
		+ (n = 92)	− (n = 39)	
Sarcopenia (T4MI, PMI)	+	40	13	0.279
	−	52	26	
Osteoporosis	+	42	10	0.032
	−	50	29	
Osteosarcopenia (T4MI PMI L1)	+	17	2	0.047
	−	75	37	

^a^χ^2^ test.

PMI, pectoralis muscle index; T4MI, T4 muscle index.

Results of multivariate logistic analyses using age, PMI, presence or absence of lung cancer, presence or absence of pulmonary disease, and presence or absence of osteoporosis as covariates are shown in Table 5. PMI (odds ratio [OR] 1.1, 95% confidence interval [CI] 1.00–1.20, p = 0.049), Lung cancer (OR 0.12, 95%CI 0.037–0.39, p < 0.001) remained prognostic factors related to FOIS.

**Table 5:** Ordinal logistic regression analyses for FOIS.

	OR	95%CI	p
PMI (cm2/m^2^)	1.1	1.00–1.20	0.049*
Lung cancer	0.12	0.037–0.39	<0.001*

## Discussion

This study aimed to elucidate the association between dysphagia and osteosarcopenia, and is the first such investigation, to the best of our knowledge. We found significantly lower FOIS scores in patients with osteosarcopenia than in those without sarcopenia and osteoporosis. Moreover, we identified PMI and presence of lung cancer as independent prognostic factors for FOIS score among patients with suspected dysphagia. A lower PMI was associated with lower FOIS scores, and PMI was significantly lower in the group with, than without, enteral feeding. Further, osteoporosis and osteosarcopenia were evident in the group with enteral feeding (p = 0.032 and 0.047, respectively).

Although, the prevalence of dysphagia was 16 to 23% in the independent older people in previous study [17], the prevalence of dysphagia was 90% in our study, the prevalence of our study was higher than that of the independent older people. It was caused by the existence of selection bias that the patient was referred to the dysphagia rehabilitation team.

In this study, 131 patients aged 65 years or older who were suspected of having dysphagia after hospitalization and who were referred to the dysphagia team were defined as sarcopenia and osteoporosis by measurement of plane chest CT using SYNAPSE. The DXA method has been widely used to measure the quality of skeletal muscle and bone, but some facilities do not have the equipment. Therefore, in some previous studies, the quality skeletal muscle and bone using CT has been evaluated [11, 18–22]. Both have been correlated with DXA, and there is a certain degree of accuracy. Moreover, in this study, inter-examiner confidence was high. In this respect, it is considered possible to use CT as an evaluation of the quality of skeletal muscle and bone.

Osteosarcopenia is a life-threatening prognostic factor as it increases susceptibility to falls and mortality not only in patients with diseases but also in healthy elderly adults [23, 24]. Here, the severity of dysphagia was worse in the group with osteosarcopenia, suggesting that osteosarcopenia affects the health status of older adults.

In our study, PMI was significantly lower in the group with, compared to without, enteral feeding, and showed a positive correlation with FOIS. In previous studies, clinical outcomes are worse when dysphagia is caused by sarcopenia [2, 25–28]. Our results were consistent with this observation.

We found that sarcopenia, osteoporosis, and osteosarcopenia did not affect the development of dysphagia, However, osteoporosis and osteosarcopenia influenced the development of enteral tube feeding, and the PMI was significantly smaller in the group with enteral feeding. These results suggest that the size of the skeletal muscle area as well as the presence or absence of osteoporosis determines whether enteral feeding is required. Moreover, PMI remained as an independent prognostic factor for FOIS score. This suggests that skeletal muscle quality is strongly associated with dysphagia.

Among older adults with osteoporosis-associated femoral and vertebral fractures, those with dysphagia had significantly lower SMI and BMD values, suggesting that both skeletal muscle and bone quality are associated with dysphagia [5]. Furthermore, a study of patients with COVID-19 revealed that a low BMD was an independent factor associated with mortality, and that it correlated with the clinical classification [22].

The present study also found a significant effect of osteoporosis in the group with enteral feeding. This concurred with previous findings.

Sarcopenia and osteoporosis did not affect the patients with originally suspected dysphagia who were undergoing dysphagia rehabilitation; therefore, FOIS scores of 1–5 would not have been significant. In this study, the presence or absence of osteoporosis contributes to the occurrence of FOIS scores of 1 and 2. Therefore, it is presumed that PMI contributes to the severity of dysphagia, and the presence or absence of osteoporosis is a factor that promotes exacerbation of severity. Therefore, it was thought that osteosarcopenia, a pathology that combines sarcopenia and osteoporosis, contributes to the development of enteral feeding.

We defined sarcopenia when both the PMI and T4MI were below the cutoff values. The prevalences were 43.5% and 41.5% in the groups with enteral feeding and dysphagia, respectively. Sarcopenia and dysphagia have been found in 16% of older patients admitted to a geriatric ward [24]. Moreover, the prevalences of sarcopenia in healthy individuals defined by the PMI and T4MI, are 10.1% and 8.7%, respectively [13]. The notably higher prevalence of sarcopenia in the present study can be attributed to the inclusion of persons aged ≥ 65 years who were referred to a dysphagia rehabilitation team.

We evaluated skeletal muscle quality using the SYNAPSE image analysis system with chest CT. The conventional gold standards (DXA and BIA) require specialized equipment, do not consider changes in water content with age, and can only assess bone and other soft tissues; therefore, issues remain regarding measurements in older patients [29]. If the quality of skeletal muscle and the degree of bone density can be measured on CT images used in routine medical care, sarcopenia and osteoporosis can be easily identified.

In this study, PMI was positively correlated with FOIS, making it an independent determinant of FOIS. Furthermore, PMI was significantly smaller in the group with enteral feeding. In short, the elements of sarcopenia affect the severity of dysphagia. Furthermore, osteoporosis contributed to the occurrence of FOIS score 1 and 2, which were defined as the enteral tube feeding group in this study. These results suggest that PMI contributes to the severity of dysphagia, and that the presence of osteoporosis is a factor that promotes the exacerbation of the severity of dysphagia. Therefore, osteosarcopenia, a combination of sarcopenia and osteoporosis, contributed to the occurrence of enternal feeding, and the FOIS score was significantly worse than that of patients without sarcopenia and osteoporosis.

This study had some limitations. It was conducted at a single hospital, and only patients referred to the dysphagia rehabilitation team were analyzed. Future investigation should include age-matched controls without dysphagia to emphasize the effects of osteosarcopenia on dysphagia.

The mechanisms through which osteosarcopenia affects dysphagia need to be elucidated to clarify whether treatments for bone, such as osteoporosis drugs, or for muscle are needed.

## Conclusion

The severity of dysphagia tended to be worse among older patients with both sarcopenia and osteoporosis who were referred to our dysphagia rehabilitation team. Such patients require frequent meal rounds, attending physicians, ward nurses, and a dysphagia rehabilitation team while under swallowing rehabilitation. These findings highlight the need to be aware of the risks of dysphagia.

## Supporting information

S1 Table Cut-off values. (TIF)

S1 Fig Measurement procedure. A) Measurement procedure of T4csa. B) Measurement procedure of PMcsa. C) Measurement procedure of L1 attenuation.
(TIF)

S1 File Patient data. (XLSX)
