# Supplementary figures and images for: Effect of osteosarcopenia on feeding status in hospitalized patients with suspected dysphagia
Source: PLoS One. 2024 Dec 19;19(12):e0315091. doi: 10.1371/journal.pone.0315091 (PMC11658479; doi:10.1371/journal.pone.0315091)

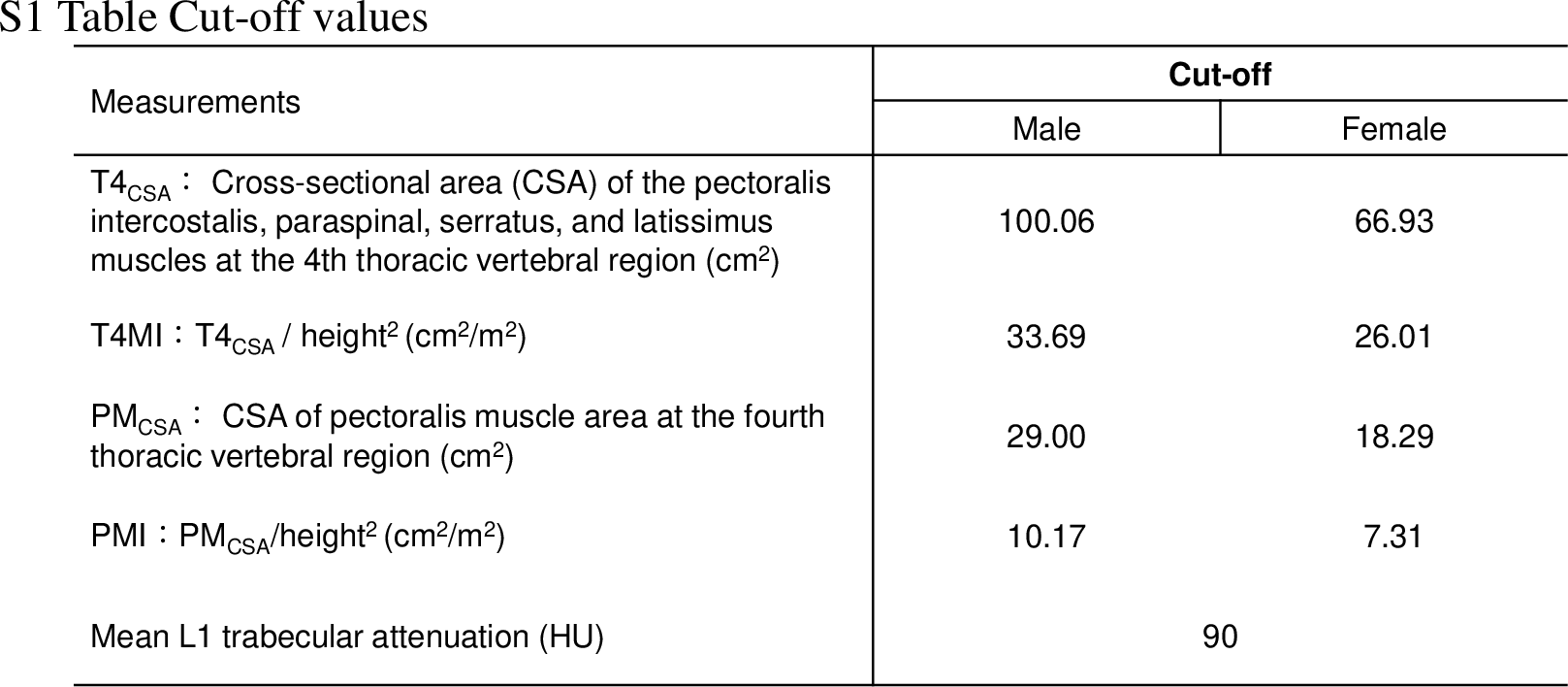

Supplement: S1 Table — (TIF) [file pone.0315091.s001.tif]

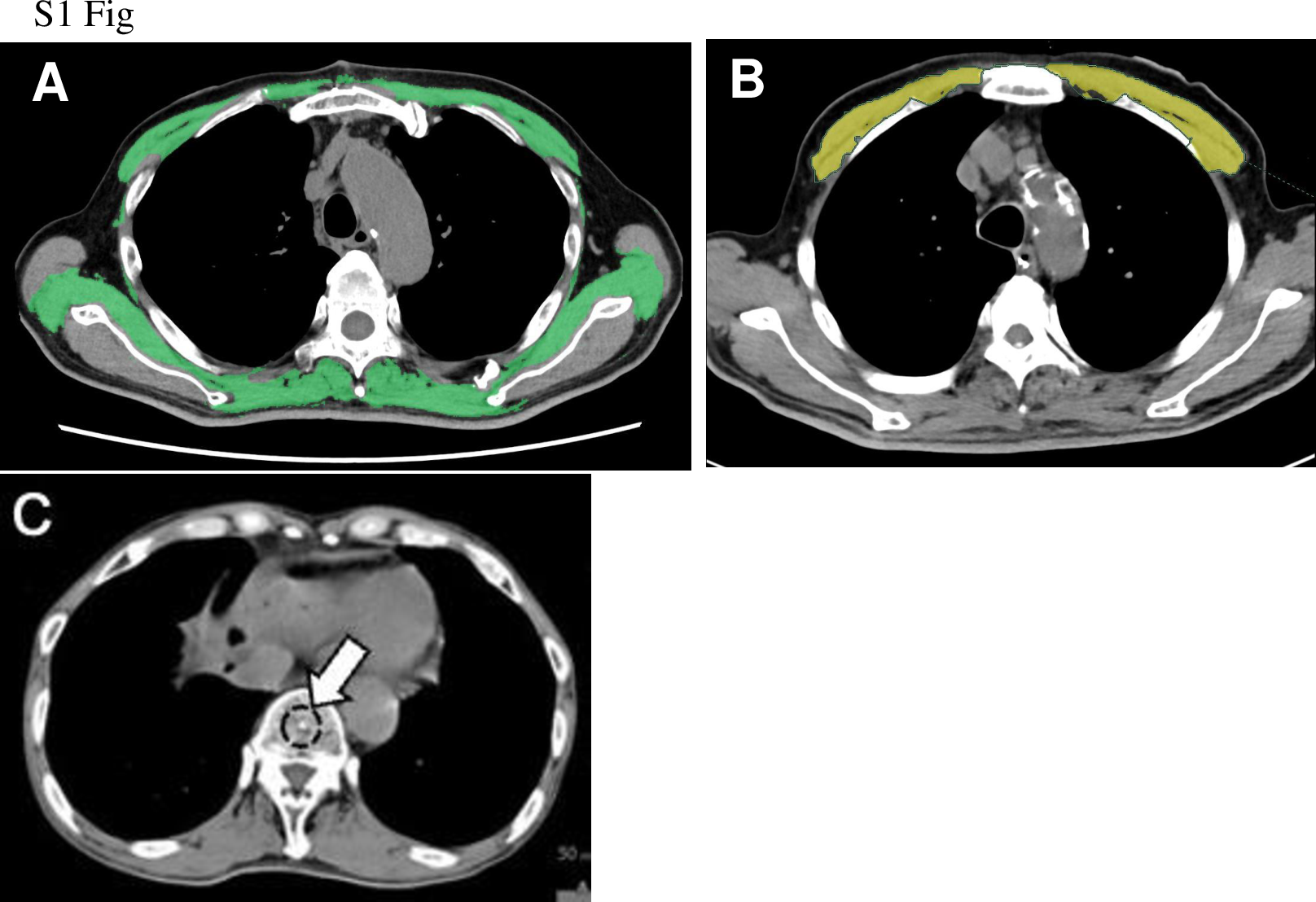

Supplement: S1 Fig — A) Measurement procedure of T4csa. B) Measurement procedure of PMcsa. C) Measurement procedure of L1 attenuation. (TIF) [file pone.0315091.s002.tif]
